# Direct Experimental Evidence for Differing Reactivity Alterations of Minerals following Irradiation: The Case of Calcite and Quartz

**DOI:** 10.1038/srep20155

**Published:** 2016-01-29

**Authors:** Isabella Pignatelli, Aditya Kumar, Kevin G. Field, Bu Wang, Yingtian Yu, Yann Le Pape, Mathieu Bauchy, Gaurav Sant

**Affiliations:** 1 Laboratory for the Chemistry of Construction Materials (LC^2^), Department of Civil and Environmental Engineering, University of California, Los Angeles, CA 90095; 2 Materials Science and Technology Division, Oak Ridge National Laboratory, Oak Ridge, TN 37861; 3 Physics of Amorphous and Inorganic Solids Laboratory (PARISlab), Department of Civil and Environmental Engineering, University of California, Los Angeles, CA 90095; 4 California Nanosystems Institute (CNSI), University of California, Los Angeles, CA 90095.

## Abstract

Concrete, used in the construction of nuclear power plants (NPPs), may be exposed to radiation emanating from the reactor core. Until recently, concrete has been assumed immune to radiation exposure. Direct evidence acquired on Ar^+^-ion irradiated calcite and quartz indicates, on the contrary, that, such minerals, which constitute aggregates in concrete, may be significantly altered by irradiation. More specifically, while quartz undergoes disordering of its atomic structure resulting in a near complete lack of periodicity, calcite only experiences random rotations, and distortions of its carbonate groups. As a result, irradiated quartz shows a reduction in density of around 15%, and an increase in chemical reactivity, described by its dissolution rate, similar to a glassy silica. Calcite however, shows little change in dissolution rate - although its density noted to reduce by ≈9%. These differences are correlated with the nature of bonds in these minerals, i.e., being dominantly ionic or covalent, and the rigidity of the mineral’s atomic network that is characterized by the number of topological constraints (n_c_) that are imposed on the atoms in the network. The outcomes have major implications on the durability of concrete structural elements formed with calcite or quartz bearing aggregates in nuclear power plants.

Concrete is used for the construction of class-I safety structures of nuclear power plants (NPPs), e.g., the containment building, biological shield and spent-fuel handling buildings, in particular. The long-term operation (LTO) of NPPs, i.e., 40 years and beyond, is expected to be affected by concrete degradation that may modify its functionality and durability^1^,^2^,^3^. A recent report of the U.S. Nuclear Regulatory Commission/Department of Energy has identified irradiation effects and alkali-silica reaction [ASR: Alkali-silica reaction is caused due to the progressive dissolution of reactive silica bearing aggregates into the concrete’s caustic pore-fluid (pH > 13). Following aggregate dissolution, a precipitate rich in alkaline elements, water and silica, i.e., an alkaline-silica hydrate (Ca_x_/Na_y_/K_z_-S_a_-H_b_, where x, y, z, a, b are coefficients) of variable composition forms. This hydrate upon formation within the concrete’s or aggregates porosity, can saturate it, resulting in deleterious cracking, and loss of mechanical properties due to internal volume expansion.] as key degradation mechanisms that require priority research in the context of license renewals and LTO of the U.S. commercial nuclear fleet^4^. In spite of several past and ongoing studies^5^,^6^,^7^,^8^,^9^,^10^,^11^,^12^,^13^,^14^, comprehension of the effects of irradiation on the mechanical and physical properties of concrete remains very limited. This is because the evolution of irradiation effects depends on several factors:

1. Damage can vary as a function of the neutron fluence and the γ-ray dosage, and also as a function of the concrete composition, i.e., the type of aggregates, and cement used and relative proportions of aggregates, water and cement in the mixture^6^,^13^,
2. Irradiation damage cannot be clearly distinguished from thermal damage caused due to absorption of radiation energy^6^,^9^, and,
3. The potential impacts of irradiation, which may render the concrete vulnerable to the other physical and/or chemical degradation processes, and vice-versa.

Mineral aggregates used in concrete are often composed of fragments of rocks including: granite and limestone, whose constituents include quartz and calcite, respectively. As per the Streckeisen diagram^15^, granites can contain between 20–60% of quartz (i.e., in terms of mineral content). Quartz is also found in other rocks such as sandstones. On the other hand, limestone is composed of calcite and minor amounts of clay and/or dolomite. Radiation damage caused to minerals such as quartz, by neutrons or, analogously, heavy-ions has been noted to be similar, suggesting similar damage mechanisms^16^,^17^. Such damage has been described by the so-called “direct impact” or “cascade” models^18^ wherein interactions between radiation and minerals causes the displacement of the primary knock-on atoms (PKAs). When these atoms have sufficient kinetic energy (K.E.), they produce subsequent “displacements cascades”^19^ within the remainder of the atomic network. This causes the formation of point defects that can return to their original lattice positions or recombine to form other defects, e.g., defect clusters, voids, dislocations, etc. When the degree of damage or amount of energy imparted into the system reaches a threshold value, amorphization localizes and then accumulates following a homogenous or heterogeneous process. This causes the disordering [“disordering” implies all actions which result in an increase in the entropy of a solid, at fixed composition. This includes the formation of defects and imperfections in initially crystalline mineral structures including the random movement of atoms to positions far removed from their original positions, due to ballistic (collision) effects. When ballistic effects dominate, a previously a previously crystalline structure, such as quartz undergoes progressive disordering until an aperiodic end-state is achieved] of the mineral’s crystal structure, resulting in a progressive loss of its structural periodicity^20^,^21^,^22^. Bylov *et al.* compiled data obtained from Russian and U.S. test reactors of the effects of irradiation temperature (from 25 °C to greater than 210 °C) on neutron radiation induced volumetric expansion of quartz for a range of amorphization levels, and for fluence levels up to about 6 × 10^20^ n/cm^2^ (E > 10 keV)^23^. An increase in the irradiation temperature was noted to shift the expansion offset to higher doses due to the annealing of point defects. Such annealing was however noted only for temperatures >200 °C. Since the operations temperature of light water reactors is limited by design to 65 °C with localized hot zones that achieve 93 °C – the annealing of defects under irradiation is expected to have little if any impact for typical nuclear power plant environments.

There is evidence in the literature that suggests that radiation-induced volumetric expansion of concrete aggregate is a major source of mechanical damage in the surrounding hardened cement paste^24^,^25^,^26^,^27^. Such mechanical damage resulting from expansion of the aggregates affects the structural properties of the concrete far more dramatically than direct irradiation effects on the cement paste itself. The effects of radiation damage on quartz and calcite (i.e., common minerals in aggregates in concrete) have been described in terms of changes in physical properties, such as density, optical properties, hardness, conductivity, etc^28^,^29^,^30^. Of these, volume change is thought to be prominent in quartz, but less significant for calcite even for neutron fluence reaching 1.0 × 10^20^ n/cm^2^ (E > 0.1 MeV)^31^,^32^. Broadly, the disordering or the amorphization of mineral aggregates is expected to result in two main effects^33^,^34^,^35^,^36^:

1. Volume changes of mineral aggregates^37^,^38^,^39^ as at fixed composition, loss of crystallinity may alter the solid density, and/or,
2. Increases in the chemical reactivity of the mineral aggregates^8^,^10^, as disordered materials are typically less chemically stable than their crystalline counterparts.

These effects would be problematic in the context of concrete durability as:

1. Increases in volume of the aggregates can cause mechanical degradation of concrete due to microcracking of the binding cement paste matrix, e.g., when the aggregate density reduces (i.e., the aggregates expand), due to a radiation induced volumetric expansion (RIVE)^13^,^14^,^39^, and,
2. Increases in aggregate reactivity could cause chemical degradation of the concrete due to the onset of irradiation-assisted alkali-silica reaction (IA-ASR); caused by the dissolution of siliceous aggregates into the caustic liquid-phase (pH > 13) contained in the concrete’s porosity^8^,^10^.

Given that the aggregates form around 70% of a concrete’s volume, their degradation would weaken the concrete, perhaps compromising the integrity of concrete elements that fulfill structural, shielding, and/or containment functions in NPPs. This has implications on the safety of NPPs in the event of reactor over-pressurization shock, or earthquakes, and, with the increasing age of NPPs, as radiation induced degradation will increase over a multi-decade service-life.

Therefore, this work elucidates how irradiation alters the atomic structures of minerals that commonly constitute aggregates in concrete. Focus is placed on contrasting how irradiation may or may not influence the chemical reactivity of a mineral with aqueous solutions, quantified in terms of its dissolution rate, in relation to chemical composition. The outcomes have significant implications on specifying radiation resistant aggregates for use in NPP concretes, and for assessing the risk-profiles of which concretes and hence nuclear power plants may be possibly more sensitive, to radiation-induced damage, than others.

## Results and Discussion

**Figure 1:**
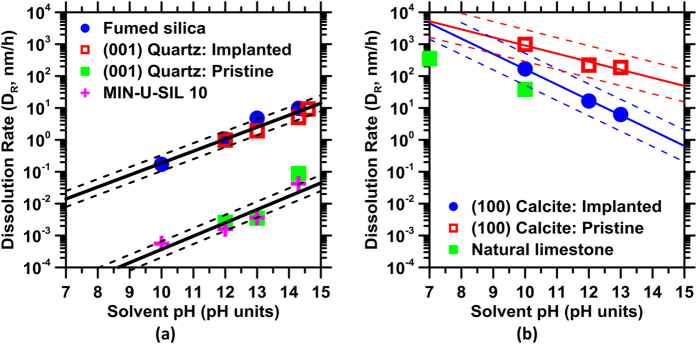
The dissolution rate (T = 25° ± 3 °C, *p* = 1 bar) as a function of solution pH for: (**a**) SiO_2_ based solids and (**b**) CaCO_3_ based solids. In the case of SiO_2_ based solids, the dissolution rate increases with pH, while the opposite is true for CaCO_3_ based solids. It is noted that, while quartz shows slight, if any, sensitivity to surface orientation, calcite dissolution appears more sensitive to surface orientation, and potentially solid composition (i.e., impurities present in the natural limestone). The thick solid lines show trends in dissolution rates while the thin dashed lines show the corresponding uncertainty bounds. The trend lines are fitted to an equation of the form: *D*_*R*_ = *A* · exp(±*B* · pH), where *A* and *B* are numerical constants. The highest uncertainty in the measured dissolution rates is on the order of ±0.5 log units.

**Figure 2:**
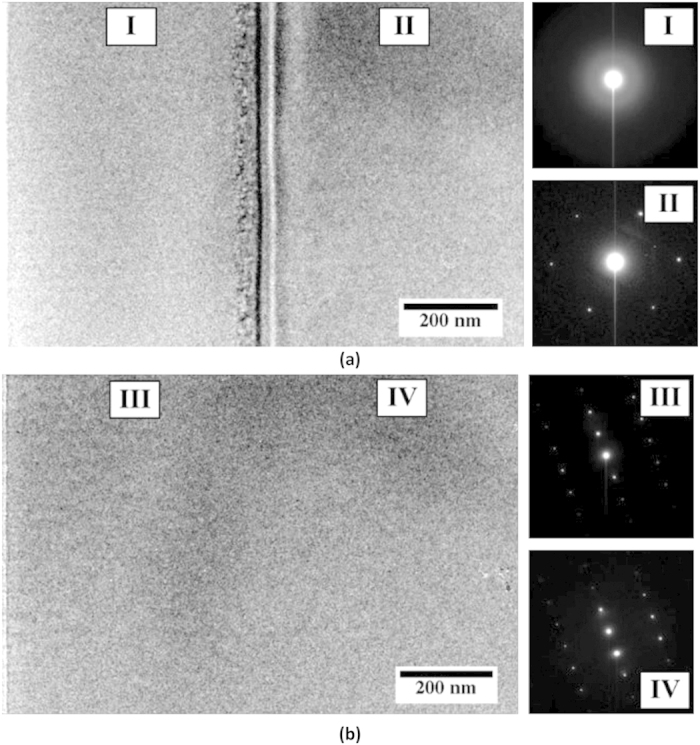
Cross-sectional TEM images and corresponding selected area electron diffraction (SAED) patterns from the ion implanted regions (I,III) and past ion end-of-range (II,IV) for: (**a**) α-quartz single crystal and (**b**) calcite single crystal. (**a**-**I**) shows the amorphization of α-quartz, while (**b**-**III**) shows the lack of amorphization in calcite. Ion implantation was carried out at an energy of 400 keV using Ar^+^-ions for a total fluence of 1.0 × 10^14^ Ar^+^/cm^2^ at room temperature. The free-sample surface is located towards the left extremity of the image(s).

Figure 1 shows the dissolution rates of α-quartz and calcite, before and following their irradiation. Also shown are the dissolution rates of pulverized quartz, fumed silica, and of natural limestone samples for comparison. It is noted that the dissolution rate of α-quartz significantly elevates after irradiation to full amorphization; attaining near equivalence to the dissolution rate of fumed (glassy) silica for the same solution compositions. This enhancement in the dissolution rate by around 3 orders of magnitude indicates that the chemical stability of α-quartz is very significantly compromised following irradiation. Calcite, on the other hand, shows a slight decrease in its dissolution rate following irradiation – even when the measurement uncertainty is accounted for (see Fig. 2b). These results, which comprise the first direct experimental evidence of differing irradiation-induced reactivity alterations, highlight a specificity to structure and composition – where quartz exhibits enhancements in aqueous reactivity, while calcite shows little if any change in reactivity following irradiation.

It should also be noted that, while minerals would show differences in their chemical reactivity (in aqueous solution) as a function of surface orientation and surface energy, this effect is more pronounced in the case of calcite than quartz. For example, the pulverized quartz (MIN-U-SIL 10), in spite of presenting a multiplicity of surface orientations, shows a dissolution rate similar to the (001)-quartz single crystal. On the other hand, the natural limestone shows more substantial differences (±0.5 log units, see Fig. 1b) in its dissolution rate, as a compared to the (100)-calcite surface. While this behavior may indeed be influenced by differences in the nature of surface defects, or elemental impurities present in the calcite samples – it is within range of experimental uncertainty, which is higher for calcite, than for quartz dissolution rates.

Near equivalence in the dissolution rates of amorphous silica, and α-quartz following irradiation suggests that the latter has been disordered under radiation exposure – a consequence which explains elevations of its dissolution rate. Dissolution has been explained within the context of crystal growth theory, focusing on the free-energy difference between the dissolving solid, and the solution in contact^40^,^41^. As such, it has been shown that dissolution occurs preferentially, and originates from high-energy sites on surfaces (structural defects and impurities), favoring the formation of etch pits (shown below). While this explanation is consistent for crystal dissolution, for compounds such as amorphous silica, the lack of atomic periodicity/structural disorder only implicates the role of (ionic) impurities in altering dissolution rates. Impurities, when present, are thought to disrupt/weaken intermolecular bonds, destabilizing a solid, either crystalline (quartz) or amorphous (silica). Such weakening ensures that a smaller driving force is sufficient to overcome the free energy barrier; thereby making both quartz and silica more susceptible to dissolution – but, only when impurities may be present. While this view is reasonable, it does not explain, or parametrize differences in the dissolution rates of compositionally analogous, and phase pure solids – e.g., quartz and amorphous silica, when they dissolve in similar solutions.

To assess structural alterations induced by irradiation, cross-sectional TEM and SAED patterns were acquired and are shown in Fig. 2. The sharp diffraction maxima noted in the SAED pattern past the end-of-range regions (i.e., of Ar^+^ implantation) of quartz (Fig. 2a(II)) are not observed in SAED patterns of the corresponding ion-irradiated regions (Fig. 2a(I)). Rather, a diffuse band is noted, which is indicative of complete amorphization. This amorphous region extends ≈550 nm into the sample, in agreement with the depth of the damage zone predicted by SRIM (≈550 nm, see Fig. S1). On the other hand, no changes in crystallinity are noted in calcite after irradiation – that is, SAED patterns of implanted (Fig. 2b(IV)) and pristine regions (Fig. 2b(III)) are similar and both present sharp diffraction maxima. Thus, while quartz attains a *metamict* state following ion-implantation, calcite remains resistant to radiation damage for the same implantation dose.

Such composition linked structural differences were also suggested in^13^, on the susceptibility of different minerals to irradiation. Evidence for such behavior is also noted in vibrational (FTIR) spectra acquired on irradiated samples which shows a shift toward lower wavenumbers and an intensity decrease of the asymmetric stretching modes (777, 1170 cm^−1^) of quartz and of the CO_3_^2−^ bending/stretching modes of calcite (712, 874 and 1350 cm^−1^). Such alterations in FTIR patterns have been attributed to structural modifications caused by irradiation including: changes in the average Si-O-Si inter-tetrahedral angle and Si-O-Si bonds in quartz^42^,^43^, and the distortion and breakage of carbonate groups in calcite^44^. Collectively, these results support the idea that, while calcite is slightly influenced by irradiation, quartz undergoes severe damage, resulting in disorder and lack of longer-range (>10 Å) periodicity.

Similar results of irradiation damage induced in calcite and quartz have also been found in natural thorium/uranium containing deposits, e.g., in: calcite crystals surrounding urano-thorianite in Tranomaro granulitic skarns^45^ and quartz found in the uranium-mineralized Athabasca Basin in Canada^46^. Seydoux-Guillaume *et al.* noted that calcite appears to be more resistant to irradiation damage than diopside (pyroxene, a silicate mineral), due to the type of interatomic forces in the former’s structure^5^. This conclusion is in agreement with data presented herein. Radiation damage of quartz was investigated by Botis *et al.* using cathodoluminescence (CL) and electron paramagnetic resonance (EPR). Their results highlight the formation of oxygen vacancy centers, silicon vacancy hole centers and peroxy centers^6^. But, in a recent study^47^, Wang *et al.* showed that measurements of damage level based simply on point defect analyses provoke only a partial view of the damage evolved. Based on these latter results, the identification of oxygen vacancy centers following radiation damage suggests that substantial modification and amorphization has already been induced in the Si-O atomic network.

**Figure 3:**
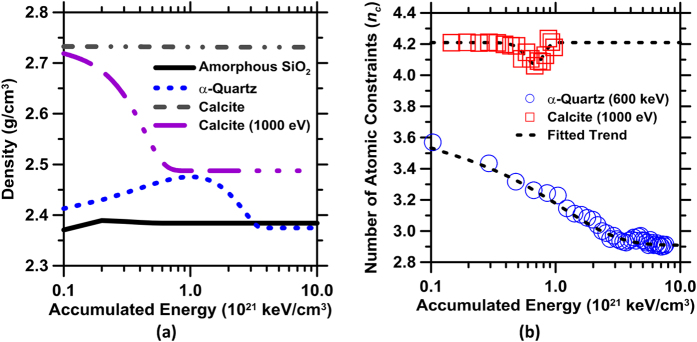
(**a**) The density evolution of pure minerals as a function of accumulated radiation dosage. The density of quartz is somewhat underestimated due to the choice of interatomic potential used in the MD-simulations^50^ and (**b**) The evolution of *n*_*c*_, in α-quartz and calcite as a function of accumulated radiation dosage. Calcite when exposed to 600 eV incident radiation remains unaffected. However, a higher incident energy of 1000 eV is noted to induce changes in density. Density changes are provoked by rotations and distortions of the CO_3_^2−^ groups with respect to the Ca-atom positions, post-irradiation. Unless stated otherwise, an incident energy of 600 eV is simulated.

Original molecular dynamics simulations^48^ show that, in agreement with the TEM-SAED analysis, that quartz is near completely disordered by radiation, while calcite is far less affected. It should be noted that the qualifier “disordered” is used in lieu of amorphized, as the resultant glassy-SiO_2_ structure formed following irradiation though non-periodic, is not equivalent to amorphous silica (Figs 2 and S2). Calcite shows substantially more resistance to radiation induced alterations. For example, for an incident energy of 600 eV no change in its structural, or physical parameters is produced. At higher incident energies, on the order of 1000 eV, calcite experiences alterations in the form of distortions and rotations of its CO_3_^2−^ groups with respect to the Ca-atom positions. These distortions and rotations of the CO_3_^2−^ groups, however, alter the atomic packing of the calcite structure – as a result of which calcite expands, resulting in a reduction in density. This expansion, which increases with the radiation dosage, achieves a limiting value, when the density of irradiated calcite stabilizes to a value of ≈90% of the pristine phase (see Fig. 3a). While this magnitude of expansion is larger than that estimated by Wong^49^ following high neutron fluence exposure, given the limited data available, this difference cannot be marked to an inconsistency of the simulation scheme.

**Figure 4:**
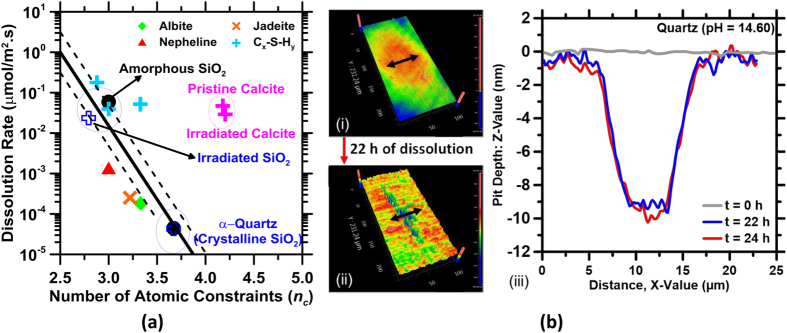
(**a**) A dissolution sensitivity diagram, which shows the dissolution rates of minerals and selected glasses as function of *n*_c_ at pH 13 (0.15 M NaOH); a concrete relevant pH (T = 25° ± 3 °C, *p* = 1 bar). Dissolution sensitivities of *more covalently bonded silicate minerals* increase with a decreasing number of atomic constraints (*n*_c_). Decreasing *n*_c_ often denotes an increase in atomic disorder, e.g., as induced by irradiation. *More ionically bonded minerals* such as calcite show little if any change in *n*_*c*_ and dissolution rates following irradiation (Fig. 1b). Rate data for glasses compositionally analogous to albite, jadeite, and nepheline was sourced from the literature^50^ while all other rate data was measured using VSI. The thick dashed lines show the trends in dissolution rates while the thin solid lines show relevant uncertainty bounds. The trend line is fitted to an equation of the form: *D*_*R*_ = *A* · exp(*B* · *n*_*c*_), where *A* and *B* are numerical constants. The highest uncertainty in the measured dissolution rate(s) is on the order of ±0.5 log units. (**b**) 3D VSI images of a (100)-surface of irradiated quartz: (i) prior to its dissolution, and (ii) after 22 hours of dissolution at pH 14.6. The cross section profile (ii) indicates the change in the morphology caused due to etch pit opening on the surface.

Due to the nature of interatomic potentials selected in the calculations, i.e., chosen to capture lattice dynamics accurately for disordered silica, but somewhat less so for α-quartz, the density of pristine quartz and of amorphous silica is underestimated. This underestimation is on the order of 7.5% for both the silicate solids, i.e., ρ ≈ 2.42 g/cm^3^ (calculated) and ρ ≈ 2.62 g/cm^3^ (measured) for α-quartz, and ρ ≈ 2.37 g/cm^3^ (calculated) and ρ ≈ 2.2 g/cm^3^ (measured) for disordered silica. Given that the terminal density of quartz following irradiation matches that of disordered silica, quartz would undergo a reduction in density, or conversely an increase in its molar volume of around 15%. This extent of volumetric expansion (swelling) is in excellent agreement with the analyses of Field *et al.* who estimated that α-quartz would swell around 14% upon its complete disordering^13^. Amorphous silica, on the other hand, shows slight, if any changes in its density^28^; around 1% across all radiation dosages (Fig. 4a). It should be noted, that irradiation induces significant changes in the inter-tetrahedral (Si-O-Si) bond angles (a decrease of around 7% is noted) in agreement with FTIR observations – but not the bond length, in irradiated quartz with respect to pristine quartz. As a result, a floppy, glassy disordered silica phase forms.

Structural disordering is not seen in calcite as, in general, as compared to the Si-O bond (E_b_ = 440 kJ/mole, where E_b_ is the bond energy) in quartz, the Ca-O bond (E_b_ = 134 kJ/mole) in calcite is weaker, and less directional in 3D (i.e., the Ca-O-Ca bond angles show a broader distribution than Si-O-Si ones, see^50^,^51^) – as a result, under radiation induced excitations – the Ca-O bond is free to reorganize, and show near complete recovery of initial (pristine) bond parameters once the radiation flux has ceased. It is postulated that this “differing behavior” is a function of the dominantly ionic character of the bonds in calcite, and the covalent character of quartz, an idea that was previously suggested by Wong^49^. This suggests that radiation perturbs the weaker angular bonds, rather than stronger radial constraints: the former which, in calcite, cannot be perturbed any further. This explains why ionically bonded solids may indeed be more resistant to radiation fluxes, than their covalent counterparts.

To comprehensively elucidate the influences of radiation on disordering the number of atomic topological constraints is computed. In solids, atoms are constrained by radial bond-stretching (BS) and angular bond-bending (BB) interactions, which act to maintain bond lengths and angles fixed around their average values. Analogous to Maxwell’s stability analysis of a mechanical truss^52^, the rigidity of a solid can be determined by enumerating the total number of constraints per atom (*n*_*c*_, unitless), and by then comparing *n*_c_ to the number of degrees of freedom per atom (i.e., three in 3D). As such, atomic networks can be classified as being flexible, i.e., hypostatic, (*n*_*c*_ < 3)^53^, showing internal low-energy modes of deformation, stressed-rigid (*n*_*c*_ > 3), i.e., being locked or hyperstatic, or being isostatic (i.e., statically determinate with *n*_*c*_ = 3). As a point of note, the hardness of such atomic structures, in order of their instability scales from: flexible, to isostatic to stressed rigid networks in ascending order, i.e., from least hard to most hard^54^,^55^.

It is noted that quartz and calcite, in the pristine state both show a stressed-rigid type character. However, following irradiation, while quartz transitions to a flexible state (i.e., *n*_*c*_ ≈ 2.9), calcite remains stressed-rigid (i.e., *n*_*c*_ ≈ 4.2, Fig. 3b). Interestingly, when the dissolution rates of these solids are cast as a function of the number of atomic constraints for a given (fixed) solution pH – a significant trend results as shown in Fig. 4(a). Specifically, in the case of SiO_2_-based solids, the dissolution rate is noted to smoothly, and linearly increase with reducing *n*_*c*_ – spanning from pristine to irradiated quartz, and from pulverized to fumed silica respectively. On the other hand, calcite, which shows no change in *n*_*c*_, independent of radiation exposure, correspondingly shows little, if any change in its dissolution rate. The slight reduction that manifests in calcite dissolution rates, for irradiated calcite, is likely due to: (a) rapid dissolution of the surface exposed to solution such that distortions of CO_3_^2−^ groups, may render their removal easier, or (b) may be related to observations of a slight increase in calcite hardness, and hence stability following irradiation^29^,^56^. If the former mechanism is operative, facilitated surface dissolution (i.e., an increase in the CO_3_^2−^ abundance in the solution, in proximity to the dissolving surface) would lower the driving force for calcite dissolution, an effect which would slightly reduce its dissolution rate (see Fig. 1b).

It should be noted that while Fig. 4(a) shows dissolution rate correlations only for pH 13 (0.15 M NaOH), the conclusions remains unchanged for other solution pH’s – an indication of the genericity of the approach – so long as the solution composition remains consistent. This diagram which indicates *the dissolution sensitivity of compositionally similar solids*, in which a specific network feature (in this case SiO_4_ tetrahedra) controls chemical instability, captures the dissolution rate dependence on *n*_c_ that is displayed by glasses compositionally analogous to albite, jadeite and nepheline^56^, and the calcium silicate hydrates (i.e., C_x_-S-H_y_, where C = CaO, S = SiO_2_, and H = H_2_O, and x and y are coefficients, where 1.2 ≤ Ca/Si ≤ 1.8, molar units), a family of disordered compounds, which comprise the primary binding and strength provisioning components of hydrated cementitious solids. These trends indicate that in regimes of high undersaturations with respect to the solute (dissolving solid), wherein defects are proposed to nucleate homogenously, the kinetics of the dissolution process can be characterized by *n*_*c*_, a fundamental indicator of the chemical instability of a solid in a solution – as a function of its atomic organization and network structure – more rigorously than other parameters, such as “the degree of crystallinity”.

## Summary and Implications on concrete durability in nuclear power plants

The outcomes of this work clarify that radiation exposure, especially in the form of heavy ions, and analogously neutrons, alters the structural, physical, and chemical properties of minerals such as calcite and quartz. While the end-effects are structural (at an atomic scale), physical and chemical in the case of quartz, they only influence the physical properties (e.g., density) of calcite. This differing behavior is correlated with the dominantly ionic nature of calcite and the covalent bonding environment in quartz, the latter of which is less resistant to radiation damage. Mineral dissolution rates are shown to be strongly correlated with the number of constraints per atom (*n*_*c*_), which describes the rigidity of a network of atoms. This offers, for the first time, a quantitative means of linking the state of atomic ordering of a given mineral to its chemical reactivity (i.e., dissolution rate). The research therefore demonstrates a basis by which chemical composition-structure-property relations can be elucidated, for pristine minerals, and for minerals which have been exposed to heat, pressure or radiation, and have thus experienced irreversible alterations of their atomic structures.

The outcomes suggest different potential routes to structural concrete damage when carbonate and silicate mineral aggregates may be exposed to radiation, e.g., when proximate to the reactor pressure vessel, in NPP environments. First, calcite on account of its expansion (i.e., its reduction in density, Fig. 3a) is expected to induce physical damage in the concrete. Such damage will result in microcracking within the binding cement paste matrix in relation to the level of (neutron) radiation exposure. However, following exposure to a threshold dosage, no further damage should evolve. Therefore, it is important to understand the implications of internal damage (microcracking) on the mechanical properties of concrete – so that its structural implications can be ascertained, and potential remediation measures implemented. Second, quartz due to its swelling, and increasing chemical instability following irradiation, is expected to expand, causing microcracking of the cement paste matrix, and eventually dissolve in the caustic cementitious pore fluid. Since any atomic disordering is expected to be progressive, quartz is expected to dissolve incrementally faster – until a metamict state is achieved, and dissolution proceeds at a limiting-rate, i.e., of glassy silica (Fig. 4a). This is problematic as the continuing dissolution of silica, so long as water, and alkali ions are available will result in the formation of an expansive alkali-silica gel, i.e., ASR. Cessation of ASR will occur only when the internal relative humidity in the concrete is lowered (RH < 80%), or when the alkalis or siliceous aggregate are consumed. This is expected to need long time scales, i.e., on the order of decades, in which time the amount of damage induced would be very substantial, and detrimental to concrete microstructure, and mechanical properties.

Both types of damage, physical and chemical are problematic as they are expected to show a gradient from the inner wall of the reactor pressure vessel (i.e., the inner surface of the reactor cavity concrete), to the outer surface of the concrete. Such gradients in expansion, i.e. strain, will result in the development of tensile hoop stresses in the reactor cavity concrete exacerbating the effects of radiation-induced damage. On a closing note, while this work has elucidated critical, and thus far unknown aspects of radiation induced alterations in minerals, significant aspects, remain worthy of evaluation: e.g., a wider range of minerals, and rocks, time dependence effects, radiation dose and energy dependence, and mechanical integrity of the affected cementitious elements. These are topics which require detailed study so that the long-term effects of radiation damage to concrete, and on NPP operations, safety and on license renewals can be rigorously and comprehensively evaluated.

## Materials and Methods

### Materials and ion-irradiations

Synthetic single crystals of α-quartz and calcite with dimensions 10 mm × 10 mm × 1 mm (l × w × h) were sourced from MTI Corporation^57^. The calcite crystals are (100)-oriented, whereas the quartz crystals are sectioned perpendicular to their optical axis (i.e., corresponding to the crystallographic *c*-axis), and are thus (001)-oriented. The quartz and calcite single-crystals were ion-beam irradiated at room temperature at the Michigan Ion Beam Laboratory (MIBL^58^) using an implantation energy of 400 keV with Ar^+^-ions to a total fluence of 1.0 × 10^14^ ions/cm^2^. No signs of blistering or significant sputtering were observed post-irradiation. The damage dose (dpa), the range and the concentrations of implanted ions were determined using SRIM using the quantification scheme proposed in^59^. In addition to the single crystals, an untreated fumed silica (Cabosil HS-5), a size graded, pulverized α-quartz (MIN-U-SIL 10) and a natural limestone were also analyzed to assess their aqueous dissolution rates, so as to establish comparisons to the oriented single crystal surfaces.

### Dissolution analysis using vertical scanning interferometry

The dissolution rates of pristine (i.e., non-implanted) and irradiated (i.e., implanted) calcite and quartz samples were measured using vertical scanning interferometry (VSI) at room temperature (25 ± 3 °C). The solutions used in these studies included: reagent grade buffer solution (pH 7, 10) and NaOH solutions prepared using deionized (DI) water: 0.015 M NaOH: pH 12, 0.15 M NaOH: pH 13, 2 M NaOH: pH 14.3, 4 M NaOH: pH 14.6. The single crystal samples were fixed onto the surface of a glass slide using an inert adhesive to facilitate handling. In the case of *flat* samples, the topographical profile of the sample mapped prior to solution contact was used as the reference plane with respect to which surface dissolution (retreat) was tracked. Powder samples were embedded in a thin-film of inert adhesive applied on the surface of a glass slide. The surface of the non-reactive adhesive once again served as the reference plane with respect to which particle dissolution was mapped^60^.

To induce solid dissolution, a small quantity of solution (i.e., 50-to-75 μL) is applied to the sample surface using a micropipette to obtain a liquid-to-solid ratio (*l/s*, mass basis) between 50,000-to-75,000, to approximate the dilute limit. This *l/s* is appropriate to minimize the effects of solution saturation, with ions, during dissolution and limit phase precipitation, if any. After allowing for a pre-determined contact time ranging between 15-to-60 minutes (i.e., depending on the mineral dissolution rates), with reapplication of the solution if needed, the solution was removed using a compressed air stream. All measurements were carried out at ambient *pCO*_*2*_. It should be noted that, in the manner implemented, the same solution, of a fixed composition repetitively contacts the mineral surface. As such, no evolutions in solution composition are permitted, and dissolution occurs at very high undersaturations with respect to the dissolving solid. As such, the dissolution rates quantified are relevant to initially fixed, essentially non-evolving solution compositions. The solution pH is the primary variable that influences the undersaturation.

A Zygo NV 9200 vertical scanning interferometer fitted with a 50× Mirau objective (N.A. = 0.55) was used in the analysis. The objective used yields a lateral spatial resolution around 500 nm. The interferometer used has a resolution of ≈0.1 nm in the vertical, i.e., *z*-direction. The analysis scheme was organized as follows: first an image of the dry sample surface, i.e., prior to solution contact was acquired. This constituted the “time-zero” (t_0_) image, and dissolution was tracked using this image, and its topographical profile (i.e., of the crystal surface, or of the particles when they are embedded in adhesive) as the reference. Following solution contact, other images were periodically acquired after the removal of the solution. These images and their topographical profiles, which were altered by dissolution, were compared to the reference image, and the change in height (Δ*h*, nm: negative in the case of dissolution which produces surface retreat) per unit time (Δ*t*, hours), reveals the solid’s dissolution rate. Each image comprises a total scanning area of 433.81 μm × 433.81 μm in stitched mode using a 3 × 3 grid, and a back-scan length of 145 μm. The total time required for capture of the full image field is on the order of 390 seconds. It should be noted that the height reduction was mapped at up to 80 discrete points on the planar single crystal, or on particle surfaces. This statistical mapping was carried out to account for the effects of surface roughness which may differ as a function of x-y (spatial) position, and may influence dissolution rates, and to ensure that the dissolution rate that were quantified account for material inhomogeneities, if any may be present. The resulting dissolution rate (*D*_*R*_, nm/h) is written as: *D*_*R*_ = (Δ*h*/Δ*t*), where *h* is the surface height (nm) for a given profile, and Δ*h* = *h*_(*i*)_ − *h*_(*i*+*1*)_ is the change in height between the successive steps measured over a dissolution period, Δ*t* (hours). It should be noted that division of *D*_*R*_ by the molar volume (*V*_*M*_, m^3^/mole) of a compound reveals the dissolution rate in units of μmol/m^2^/s. All measurements were repeated 3 times.

### Transmission electron microscopy

Cross-sectional lift-outs were prepared from pristine and irradiated quartz and calcite samples using a FEI Quanta 200i DualBeam focused ion beam (FIB). Low-angle, low-energy milling was carried out following the primary thinning to obtain electron transparent sections, while minimizing any damage that may be induced by the ion-beam. The electron transparent samples were then analyzed using a Philips CM200 transmission electron microscope (TEM) at an accelerating voltage of 200 kV. Selected area electron diffraction (SAED) patterns were acquired on both pristine and irradiated regions to determine the degree, if any, of disordering in the irradiated regions. Due care was taken to minimize the electron dose to the imaged areas to reduce damage imparted by the imaging electron beam.

### Molecular dynamics simulations

Molecular dynamics simulations were carried out on calcite and quartz structures using LAMMPS^61^ at 300 K to study the influences of radiation damage on atomic structures^47^. The simulated system consists of a supercell of pristine quartz or calcite containing between 4500 and 21000 atoms, depending on the incident irradiation energy. To simulate ballistic collisions induced by irradiation, a randomly selected atom is accelerated with a given kinetic energy (similar to an incident energy) to mimic energy transfer between radiation and an atom. The acceleration initiates a cascade of collisions between atoms, causing damage to the crystal structure. This process is repeated until the desired dosage is achieved. Quartz and calcite are simulated using the inter-atomic potentials of 62 and 63 respectively, which have been shown to suitably reproduce crystalline and amorphous structures of these two minerals^47^,^63^,^64^,^65^,^66^,^67^,^68^. In order to provide a realistic prediction of high-energy events, which cause atoms to temporarily come unusually close to each other, the Ziegler, Biersack and Littmark (ZBL) potentials are used at short inter-atomic separations (<1 Å)^69^. Since such a small inter-atomic separation is not observed during typical conditions, i.e., close to equilibrium, the ZBL potentials only take effect during the collision cascade, while the rest of the relaxation dynamics remain unaffected. After the radiation damage simulation is completed, a series of atomic configurations are extracted at different dosage levels for detailed structural analyses. Special focus is placed on quantifying the type and nature of damage, including amorphization, and evaluating structural rigidity^70^,^71^ by analysis of atomic trajectories. The rigidity analysis consists of an enumeration of the number of intact and broken radial and angular bond constraints at 300 K^72^,^73^. A bond constraint is considered to be broken if the relative variation in the bond distance (or bond angle) is sufficiently large, i.e., exceeding 7%^50^, which indicates the absence of an underlying restoring force that would maintain the bond length (and angle) fixed around its average value. It should be noted that the constraints enumeration procedure does not significantly depend on the choice of this threshold, similar to the Lindemann criterion^50^.

## Additional Information

**How to cite this article**: Pignatelli, I. *et al.* Direct Experimental Evidence for Differing Reactivity Alterations of Minerals following Irradiation: The Case of Calcite and Quartz. *Sci. Rep.* **6**, 20155; doi: 10.1038/srep20155 (2016).

## Supplementary Material

Supplementary Information
