# Supplementary material for: Direct Experimental Evidence for Differing Reactivity Alterations of Minerals following Irradiation: The Case of Calcite and Quartz
Source: Sci Rep. 2016 Jan 29;6:20155. doi: 10.1038/srep20155 (PMC4731743; doi:10.1038/srep20155)
Supplement: Supplementary Information [file srep20155-s1.pdf]

Supplementary Information

Manuscript title: Direct Experimental Evidence for Differing Reactivity Alterations of Minerals following Irradiation: The Case of Calcite and Quartz

Authors List: Isabella Pignatelli, Aditya Kumar, Kevin G. Field, Bu Wang, Yingtian Yu, Yann Le Pape, Mathieu Bauchy, Gaurav Sant

The influence of solution composition on quartz and calcite dissolution rates

The dissolution rate of quartz increases with pH while that of calcite decays. This pH dependence of quartz dissolution has been previously explained in terms of: the undersaturation of the bulk solution, the speciation of silicon, and the surface complexations of species ([1,2,3,4,5]). For pH > 6, negatively charged silicate species ($H_2SiO_4^{2-}$, H_3SiO^{4-}) are present in solution and protonated "≡SiO-Na^+" and de-protonated "≡SiO^-" species are present on the solid silicate surfaces. The latter species are thought to be amenable to rapid silica dissolution. It has been argued that when alkali cations, e.g., Na^+ or K^+, are present in the bulk solution, they cause a redistribution of the surface complexes, such that ≡SiO-Na^+ or ≡SiO-K^+ become more dominant than ≡SiO^- species, further enhancing quartz dissolution ([1,2,3,4,5,6,7,8,9]). Such increases in silicate dissolution rates in the presence of alkaline salts, e.g., NaCl, KCl, $CaCl_2$, $MgCl_2$, is termed as the "salt effect" ([1,9,10,11,12]), wherein the reactivity enhancement induced by alkaline cations follows an order similar to a Hoffmeister series where: $Ba^{2+} > Sr^{2+} > K^+ > Na^+ > Mg^{2+} > Li^+$ for amorphous silica and $Ba^{2+} > K^+ \approx Na^+ \approx Li^+ > Ca^{2+} > Mg^{2+}$ for α-quartz ([3,6]).

Calcite dissolution at low pH is proportional to the [H^+ and CO_3^{2-}] abundance and is impacted by mass transfer (i.e., diffusion of ions from the solid surface into the bulk solution) ([13,14,15,16,17]). On the other hand, at neutral to alkaline conditions, calcite dissolution is pH-independent and controlled by the presence of high energy sites on the surface, i.e., interface control ([18,19,20,21]). The presence of these sites is revealed by the formation of etch pits during dissolution. Pits nucleate rapidly far from solute equilibrium. As such, while 2D pits nucleate spontaneously even in defect-free areas at very high undersaturations, defect-assisted nucleation (i.e., that which is associated with structural/impurity defects) operates at intermediate undersaturation levels, with progressive step-retreats controlling dissolution rates nearer to equilibrium ([22]). On $\{10\bar{1}4\}$ surfaces, long-lived, larger and pointed pits have been attributed to the presence of line defects, while short-lived, smaller and flat-bottom pits have been attributed to point defects ([19,23]). These pits show a rhombic morphology ([14,19,21,23,24,25,26]), though pyramidal pits may also form on the $\{10\bar{1}0\}$ surfaces (100), as is seen in the present study (not shown). Similar to silica-based minerals, the dissolution of calcite is also affected by the presence of divalent cations ([21]). At neutral to basic pH, Ca^{2+}, Mg^{2+}, Sr^{2+} and Ba^{2+} ions and metals such as Cd, Ni, Cu, Co and Mn inhibit calcite dissolution due to interactions with its surface ([25,26,28,29,30,31,32,33,34,35,36,37,38]).

References

[1] Dove, P. M. & Crear, D. A. Kinetics of quartz dissolution in electrolyte solutions using a hydrothermal mixed flow reactor. *Geochim. Cosmochim. Acta* **54**, 955-969 (1990).
[2] Seward, T. M. Determination of the first ionization constant of silicic acid from quartz solubility in borate buffer solutions to 350°C. *Geochim. Cosmochim. Acta* **38**, 1651–1664 (1974).
[3] Plettinck, S., Chou, L. & Wollast, R. Kinetics and mechanism of dissolution of silica at room temperature and pressure. *Mineral. Mag.* **58**, 728-729 (1994).
[4] House, W. A. The role of surface complexation in the dissolution kinetics of silica: effects of monovalent and divalent ions at 25°C. *J. Colloid Interface Sci.* **163**, 379-390 (1994).
[5] Dove, P. M. The dissolution kinetics of quartz in sodium chloride solutions at 25°C to 300°C. *Am. J. Sci.* **294**, 665-712 (1994).
[6] Dove, P. M. & Nix, C. J. The influence of the alkaline earth cations, magnesium, calcium and barium on the dissolution kinetics of quartz. *Geochim. Cosmochim. Acta* **61**, 3329–3340 (1997).
[7] Dove, P. M. The dissolution kinetics of quartz in aqueous mixed cation solutions. *Geochim. Cosmochim. Acta* **63**, 3715–3727 (1999).
[8] Icenhower, J. P. & Dove, P. M. The dissolution kinetics of amorphous silica into sodium chloride solutions: effects of temperature and ionic strength. *Geochim. Cosmochim. Acta* **64**, 4193–4203 (2000).
[9] Dove, P. M., Han, N. & De Yoreo, J. J. Mechanisms of classical crystal growth theory explain quartz and silicate dissolution behavior. *Proc. Natl. Acad. Sci.* USA **102(43)**, 15357–15362 (2005).
[10] Diénert, F. & Wandenbulke, F. Sur le dosage de la silice dans les eaux. *C. R. Séances Acad. Sci.* **176**, 1478–1480 (1923).
[11] van Lier, J. A., de Bruyn, P. L. & Overbeek, T. G. The solubility of quartz. *J. Phys. Chem.* **64**, 1675-1682 (1960).
[12] Dove, P. M., Han, N., Wallace, A.F. & De Yoreo, J. J. Kinetics of amorphous silica dissolution and the paradox of the silica polymorphs. *Proc. Natl. Acad. Sci*. **105(29)**, 9903–9908 (2008).
[13] Rickard, D. & Sjöberg, E. L. Mixed kinetic control of calcite dissolution rates. *Am. J. Sci.* **283**, 815-830 (1983).
[14] Shiraki, R., Rock, P. A. & Casey, W. H. Dissolution kinetics of calcite in 0.1 M NaCl solution at room temperature: an atomic force microscopic (AFM) study. *Aquat. Geochem.* **6**, 87–108 (2000).
[15] Sjöberg, E. L. & Rickard, D. T. Calcite dissolution kinetics: surface speciation and the origin of the variable pH dependence. *Chem. Geol.* **42**, 119-136 (1984).
[16] Sjöberg, E. L. & Richard, D. T. The effect of added dissolved calcium on calcite dissolution kinetics in aqueous solutions at 25°C. *Chem. Geol*. **49**,405-413 (1985).
[17] Compton, R. G. & Pritchard, K. L. The dissolution of calcite at pH > 7: kinetics and mechanism. *Philos. Trans. R. Soc. Lond. Ser. Math. Phys. Sci.* **330(1609)**, 47–70 (1990).
[18] Schott, J., Brantley, S., Crear, D., Guy, C., Borcsik, M. & Willaime, C. Dissolution kinetics of strained calcite. *Geochim. Cosmochim. Acta* **53**, 373–382 (1989).
[19] MacInnis, I. N. & Brantley, S. L. The role of dislocations and surface morphology in calcite dissolution. *Geochim. Cosmochim. Acta* **56**,1113–1126 (1992).
[20]Van Cappellen, P., Charlet, L., Stumm, W. & Wersin, P. A surface complexation model of the carbonate mineral-aqueous solution interface. *Geochim. Cosmochim. Acta* **57**, 3505–3518 (1993).
[21] Atanassova, R., Cama, J., Soler, J. M., Offeddu, F. G., Queralt, I. & Casanova, I. Calcite interaction with acidic sulphate solutions: a vertical scanning interferometry and energy-dispersive XRF study. *Eur. J. Mineral.* **25**, 331–351 (2013).

[22] Teng, H. H. Controls by saturation state on etch pit formation during calcite dissolution. *Geochim. Cosmochim. Acta* **68**, 253–262 (2004).
[23] Liang, Y., Baer, D. R., McCoy, J. M., Amonette, J. E. & LaFemina, J. P. Dissolution kinetics at the calcite-water interface. *Geochim. Cosmochim. Acta* **60**, 4883–4887 (1996).
[24] Duckworth, O. W. & Martin, S. T. Dissolution rates and pits morphologies of rhombohedral carbonate minerals. *Am. Mineral.* **89**, 554-563 (2004).
[25] Vinson, M. D., Arvidson, R. S. & Lüttge A. Kinetic inhibition of calcite (104) dissolution by aqueous manganese (II). *J. Cryst. Growth* **307**, 116-125 (2007).
[26] Xu, M. & Higgins, S. R. Effects of magnesium ions on near-equilibrium calcite dissolution: step kinetics and morphology. *Geochim. Cosmochim. Acta* **75**,719–733 (2011).
[27] Thomas, J.M. & Renshaw, G. D. Dislocations in calcite and some of their chemical consequences. *Trans. Faraday Soc.* **61**, 791–796 (1965).
[28] Morse, J. W. & Arvidson, R. S. The dissolution kinetics of major sedimentary carbonate minerals. *Earth Sci.Rev*. **58**, 51-84 (2002).
[29] Ardvinson, R. S., Collier, M., Davis, K. J. & Vinson, M. D. Magnesium inhibition of calcite dissolution kinetics. *Geochim. Cosmochim. Acta* **70**, 583-594 (2006).
[30] Lea, A. S., Amonette, J.E., Baer, D. R., Liang, Y. & Colton, N. G. Microscopic effects of carbonate, manganese and strontium ions in calcite dissolution. *Geochim. Cosmochim. Acta* **65**, 369-379 (2001).
[31]Sjöberg, E. L. Kinetics and mechanism of calcite dissolution in aqueous solutions at low temperatures. *Stockholm Contrib. Geol.* **32**, 32 (1978).
[32] Buhmann, D. & Dreybrodt, W. Calcite dissolution kinetics in the system H_2O-CO_2-$CaCO_3$ with participation of foreign ions. *Chem. Geol*. **64**, 89-102 (1987).
[33] Gytjahr, A., Dabringhaus, H. & Lacmann, R. Studies of the growth and dissolution kinetics of the $CaCO_3$ polymorphs calcite and aragonite: II the influence of divalent cation additives on the growth and dissolution rates. *J. Cryst. Growth* **158**, 310-315 (1996).
[34] Salem, M. R., Mangood, A.H. & Hamdona, S. K. Dissolution of calcite crystals in the presence of some metal ions. *J. Mater. Sci*. **29**, 6463-6467 (1994).
[35] Martin-Garin, A., Van Cappellen, P. & Charlet, L. Aqueous cadmium uptake by calcite: a stirred flow-through reactor study. *Geochim. Cosmochim. Acta* **67**, 2763-2774 (2003).
[36] Plummer, L. N. & Mackenzie, F. T. Predicting mineral solubility from rate data – application to the dissolution of magnesium calcites. *Am. J. Sci.* **274**, 61-83 (1974).
[37] Stoller, R. E., Toloczko, M. B., Was, G. S., Certain, A. G., Dwaraknath, S. & Garner, F. A. On the use of SRIM for computing radiation damage exposure. *Nucl. Instrum. Meth. B* **310**, 75–80 (2013).
[38] Humphrey, W., Dalke, A. & Schulten, K. VMD: visual molecular dynamics. *J. Mol. Graphics* **14**, 33-38 (1996).

Supplementary Figure Legends

Figure S1: The calculated damage dose (dpa) and implanted ion concentration (appm) as a function of depth into the sample for calcite and α-quartz. These values are calculated using the scheme implemented in ([94]) for an implantation energy of 400 keV using Ar^+-ions for a total fluence of 1.0 x 1014 Ar+/cm^2. Ion implantation was carried out at room temperature and the free-sample surface is located at "x = 0 nm" – towards the left extremity of the plot. As a function of their similar density pre-irradiation, Ar^+ ions are implanted to a depth of 550 nm in both minerals.

Figure S2: (a) The progressive disordering of α-quartz to disordered silica under energetic particle exposure (Left image: red: oxygen atoms, yellow: silicon atoms) and (b) The evolution of the atomic structure of calcite following irradiation. Structural evolution in calcite manifests only in the form of CO_3^{2-} groups experiencing random rotations and distortion in the irradiated structure (Right image: blue: calcium atoms, red: oxygen atoms, brown: carbon atoms). The structural data were sourced from molecular dynamics (MD) simulations and visualized using VMD ([95]).

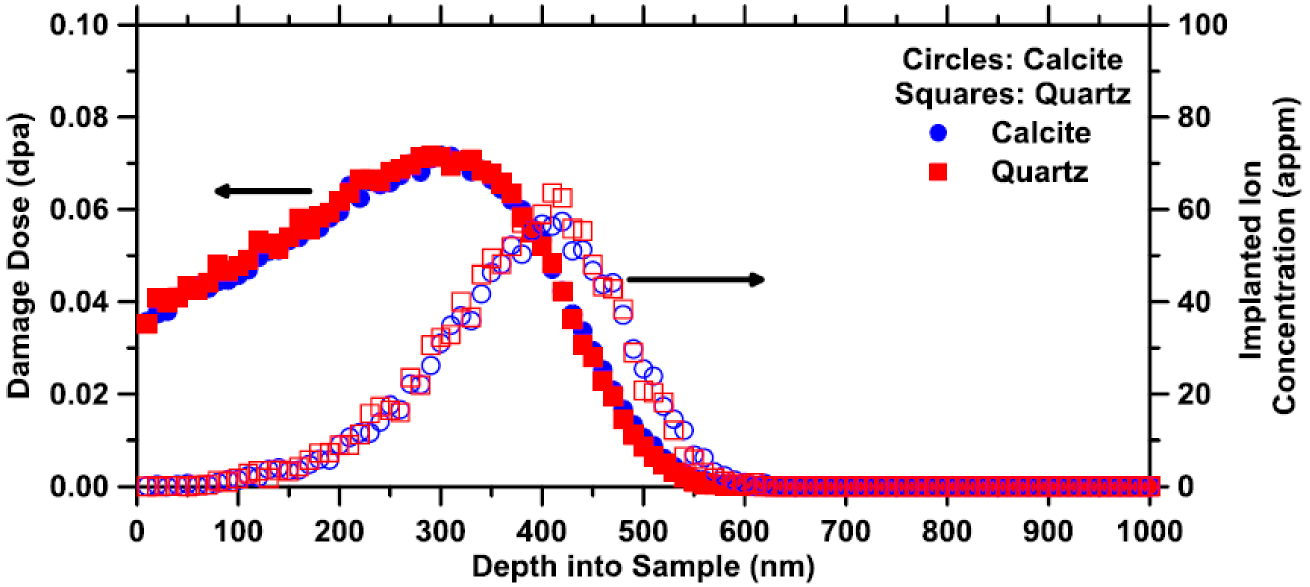

Figure S1

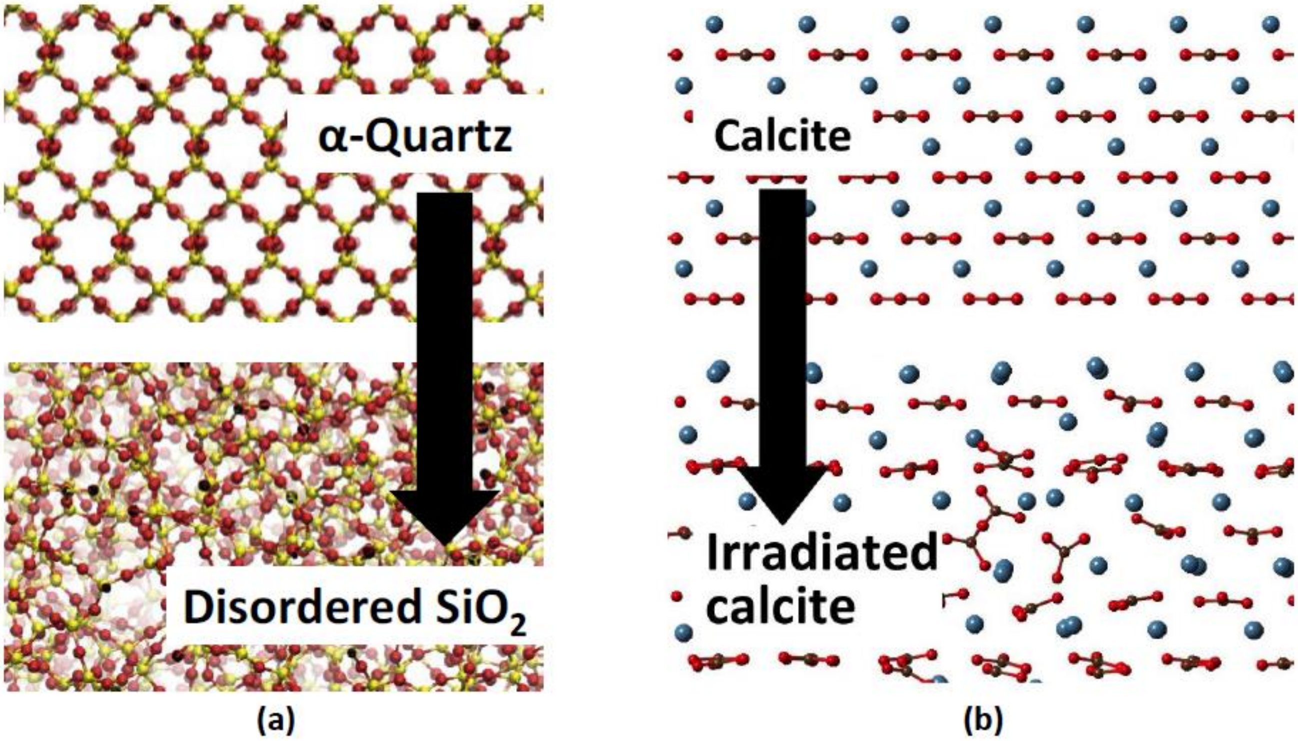

Figure S2
